# Metachondromatosis: A Confusing Disease

**DOI:** 10.1055/s-0041-1736614

**Published:** 2021-11-04

**Authors:** Alejandro Blasco, Marta Salom, Francisco Giner, Emilio Baixauli, Francisco Baixauli

**Affiliations:** 1 Membro Superior e Unidade Nervosa Periférica, Hospital Universitário Politécnico de La Fé, Valência, Espanha; 2 Ortopedia Pediátrica e Unidade de Trauma, Hospital Universitário Politécnico de La Fé, Valência, Espanha; 3 Patologia Anatômica, Hospital Universitário Politécnico de La Fé, Valência, Espanha; 4 Tumores Musculoesqueléticos e Unidade de Infecção Articular, Hospital Universitário Politécnico de La Fé, Valência, Espanha

**Keywords:** bone neoplasms/pathology, child, enchondromas, exostoses, multiple hereditary, osteochondromas

## Abstract

Metachondromatosis is a rare autosomal dominant genetic disease with incomplete penetrance that involves abnormal function of the *PTPN11* gene. Differentiation between chondrogenic tumors is a challenge for orthopedists. We report a case of a 5 year-old girl with metachondromatosis, a disease that shares attributes with osteochondromas and enchondromas. We found multiple osteochondroma-like lesions with the atypical characteristic of guiding its growth toward the neighboring joint (epyphisis) instead of moving away from it. Furthermore, columnar enchondroma-like lesions were clearly visible in the right distal radius, in the proximal femoral cervix and in the iliac crests. The patient reported that some other tumor had disappeared or downsized with time. This case was debated between a multidisciplinary skeletal dysplasia group. The aforementioned clinical and radiographic findings reinforced the hypothetical diagnosis of metachondromatosis. Definitive diagnosis of metachondromatosis requires a combination of clinical, radiographical and histopathological findings. Differential diagnosis between enchondromas, osteochondromas and metachondromatosis is vital due to differences in malignization and natural history. When a patient has multiple enchondromas and osteochondromas with regression of some lesions and atypical radiographical characteristic of the osteochondroma-like lesions pointing toward the epiphysis, metachondromatosis, a rare disease, must be considered. Surgical treatment is reserved for painful lesions Risk of malignization is insignificant and genetic advice must be given due it is an autosomal dominant disease.

## Introduction

Osteochondromas are the most common benign cartilaginous tumor. Osteochondromas are typically metaphyseal tumors of the long bones (proximal humerus, tibia, and distal femur). They usually grow as pedunculated or sessile lesions composed of cortical tissue and with medullary bone tissue covered by a cartilaginous cap. 1

Enchondromas are the second most common benign cartilaginous tumor after osteochondroma. They are commonly found within the medullary cavity of the bones of the appendicular skeleton (more frequent in the hands than in the feet, particularly in the phalanges) and they are characterized by the formation of mature hyaline cartilage in the medullar cavity. 2

Metachondromatosis is a rare autosomal dominant genetic disease with incomplete penetrance that involves abnormal function of the *PTPN11* gene. 1

Differentiation between chondrogenic tumors is a challenge for orthopedists. We report the case of a patient with metachondromatosis, a disease that shares attributes with osteochondromas and enchondromas.

## Case Report

A 5-year-old girl was referred to our Pediatric Orthopedics Unit asking for evaluation of multiple osteochondromas. Physical examination revealed multiple painful tumor compatible with osteochondromas on radiography. Impairment for proximal interphalangeal (PIP) and distal interphalangeal (DIP) flexion of the fourth finger of the right hand was evidenced, which correlated with a middle phalanx osteochondroma. Previous history of skeletal hereditary diseases could not be confirmed. Surgical excision was performed without complications.

**Fig. 1:**
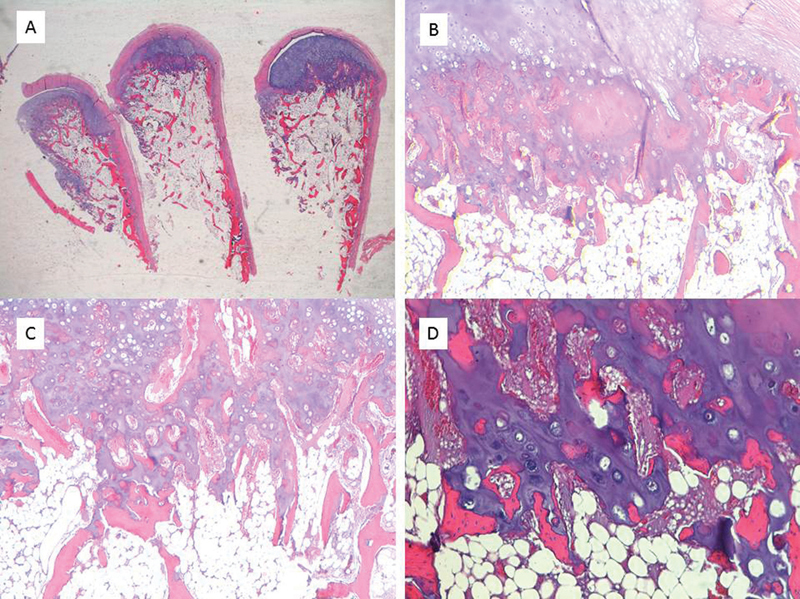
Images of different histological sections stained with hematoxylin-eosin. A) Sample of the exostotic lesion constituted by a cartilaginous cap with an osteoid central trabecular matrix, 2.5X. B) The chondral matrix shows mature characteristics with endochondral ossification, 4X. C) Transition zone between the peripheral cartilaginous component and trabecular bone resembling a slightly disorganized growth plate, 4X. D) Chondrocytes are arranged in isogenic groups, larger in the central portion, without atypia or atypical mitoses, 10X.

However, an anatomopathological examination showed multiple osseous and cartilaginous pieces compatible with the outer cap of a benign enchondroma. Eight months later, she was also operated for a growing and painful osteochondroma in the third left metacarpal and in the fourth right metacarpal. Paradoxically, in this case, the anatomopathological examination showed a 2.3 × 2 cm osseous lesion covered with a pearly-white smooth cap compatible with benign osteochondroma ( Fig. 1 ).

**Fig. 2:**
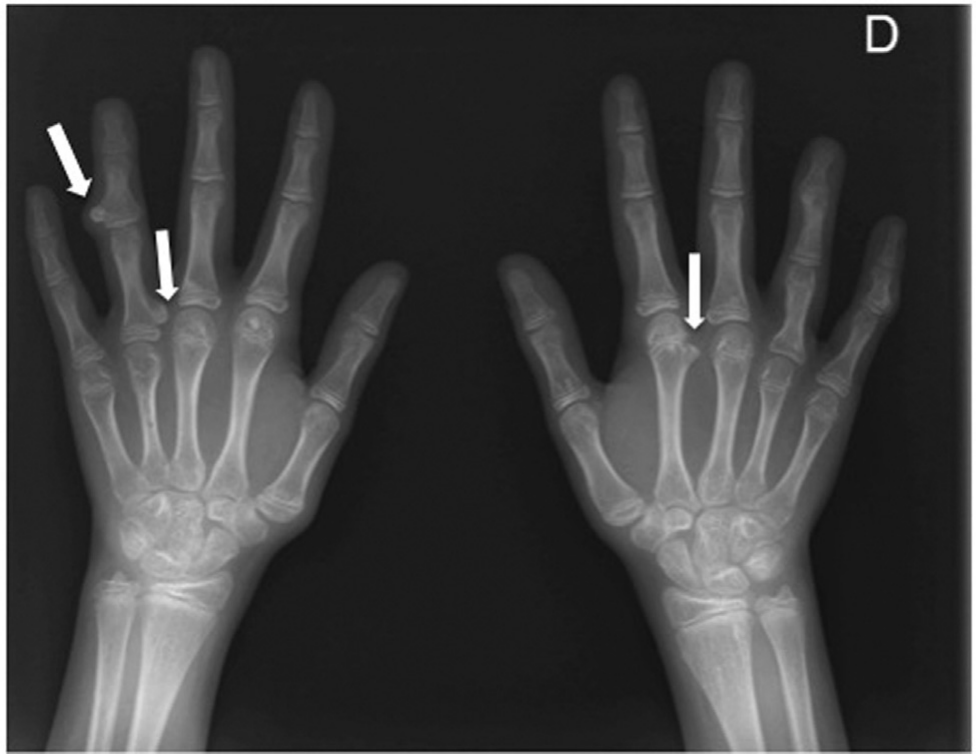
Anteroposterior view of both hands showing multiple osteochondroma-like lesions with the atypical characteristic of guiding their growth toward the neighboring joint (epyphisis) instead of moving away from it.

**Fig. 3:**
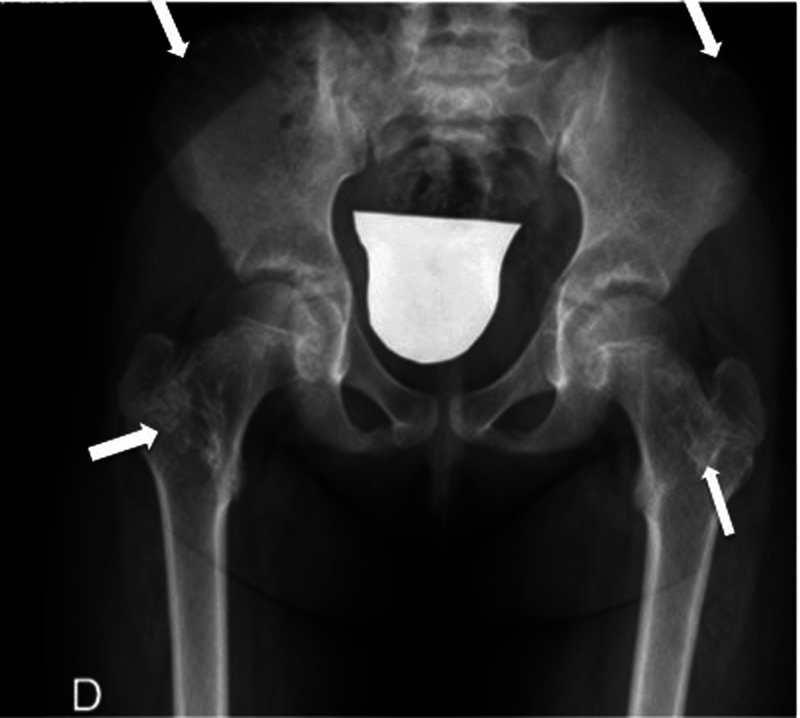
Anteroposterior view of the pelvis. In metachondromatosis, enchondromas distribute mainly around the iliac crest and metaphyseal regions of the long bones.

Previous radiographies were examined to find out a reason for this unexpected paradox. What we found was multiple osteochondroma-like lesions with the atypical characteristic of guiding its growth toward the neighboring joint (epiphysis) instead of moving away from it. Furthermore, columnar enchondroma-like lesions were clearly visible in the right distal radius, in the proximal femoral cervix and in the iliac crests ( Figs. 2 and 3 ). The patient reported that some other tumor had disappeared or downsized with time. This case was debated between a multidisciplinary skeletal dysplasia group. The aforementioned clinical and radiographic findings reinforced the hypothetical diagnosis of metachondromatosis.

After 8 years of follow-up, the patient is 13 years old, and new lesions in the right ankle, the hip, and the middle phalanx or the fourth left finger have grown, while others have regressed. However, she is asymptomatic, and she leads a normal life.

## Discussion

Metachondromatosis combines multiple metaphyseal juxtaepiphyseal exostoses, metaphyseal enchondromas, periarticular calcifications, and frequent unilateral or bilateral Legg-Calvé-Perthes-like changes in the femoral head resembling osteonecrosis. 3 4 5

Classification: Metachondromatosis is a subtype of enchondromatosis without spinal affection, autosomal dominant transmission and osteochondroma-like lesions. 6

Etiology: Metachondromatosis is related with genetic abnormalities. Fisher et al. found 31 cases published. 1 Mutation of the *PTPN11* gene (protein tyrosine phosphatase nonreceptor type 11) and lack of production of the tyrosine phosphatase SHP2 is related with the pathogenesis of metachondromatosis, as well as of other developmental diseases (Noonan syndrome, Noonan syndrome with multiple lentigines) and malignant diseases (juvenile myelomonocytic leukemia). 7 Mutation of the *PTPN11* gene is inherited in an autosomal dominant pattern with incomplete penetrance and parents must be advised of it. Unlike enchondromatosis, EXT-1 and EXT-2 mutation (exostosin protein) is not observed in metachondromatosis. 1 7

The definitive diagnosis of metachondromatosis requires a combination of clinical, radiographical, and histopathological findings ( Table 1 ). 8

**Table 1:** Differential diagnosis between enchondromas, osteochondromas and metachondromatosis

	Enchondromas	Osteochondromas	Metacondromatosis
Frequency	10% of benign osseous tumors. 8 The prevalence of Ollier disease is 1/100,000. 1	20–50% of all benign bone tumors and 10–15% of all bone tumors. 7 The prevalence of multiple osteochondromas is estimated to be 2/100,000. 1	< 1/1,000,000, < 30 cases described. 2
Location	Frequently found in the hands more than in the foot and ankle bones, particularly in the phalanges. 1	Proximal humerus, tibia, and distal femur. 1	Enchondroma-like lesions: Metaphyseal regions of the long bones and iliac crest Osteochondroma-like lesions are mainly distributed in the hands and feet. 6
Genetics	Does not follow a clear Mendelian transmission pattern	HMO is an autosomal dominant inherited trait. 7 EXT-1 and EXT-2 mutation (exostosin protein; endoplasmic reticulum transmembrane glycosyltransferase necessary for the heparan sulfate synthesis and physeal growth)	Autosomal dominant *PTPN11* gene mutation, lack of tyrosine phosphatase SHP2
Radiology	Formation of hyaline cartilage in the medulla of a bone. 2 Well-defined, expansile, lytic lesions with varying degrees of stippled or punctate calcifications in the diaphysis or metaphyseal-diaphyseal regions of the bone. 1	Cartilage pedunculated or sessile lumps outside the metaphyseal region of the long bones. 2	Epiphyseal-pointing osteochondroma-like lesions combined with calcified enchondroma-like lesions ( Figs. 1 and 2 ). They can spontaneously regress. 3
Anatomopathological examination	On gross visual inspection, an enchondroma will appear as a bluish, semitranslucent, hyaline cartilage with a distinctly lobular arrangement. These lobules will vary from a few millimeters to a few centimeters in diameter. Cytologically, an enchondroma will appear as small chondrocytes that lie in the lacunar spaces, with a small, round, regular nucleus, and no significant atypia. No mitoses will be seen. Occasional binucleate cells will be seen. Some enchondromas can contain foci of ossification within this cartilage 1	Bony lesion covered with a pearly-white smooth cap	42% as osteochondromas, 33% as enchondromas, 17% combined. 1
Natural history	New lesions do not appear after skeletal maturation	New lesions do not appear after skeletal maturation	New lesions do not appear after skeletal maturation
Malignization	5% in solitary enchondromas, >20% multiple enchondromatosis. 1	Between 0.4% and 2% in patients with solitary osteochondroma and between 1 and 4% in patients with HMO. 7	No malignization

Clinical findings: The combination of multiple enchondromas and osteochondromas raises suspicion of metachondromatosis. 3 4 Metachondromatosis has characteristically epiphyseal-pointing osteochondroma-like lesions that can spontaneously regress, in contrast with conventional osteochondromas. 3 4

Radiographical findings: In metachondromatosis, enchondromas distribute mainly around the iliac crest and the metaphyseal regions of the long bones ( Fig. 3 ). In contrast, osteochondroma-like lesions are mainly distributed in the hands and feet ( Fig. 2 ). 6 In our case, we saw that these lesions can distribute in both the axial skeleton (pelvis, spine, scapula, and hip) and the appendicular skeleton (hands and feet). The hands were the most frequently affected locations in our case, which is in line with Fisher et al. 1 Metachondromatosis is not related with shortening and deformity of the long bones, a common feature of hereditary multiple exostosis. 4 As with osteochondromatosis and enchondromatosis, new lesions do not appear after skeletal maturation. 1

Histopathological findings: Histopathological examination reported first multiple osseous and cartilaginous pieces compatible with the outer cap of a benign enchondroma and, second, a bony lesion covered with a pearly-white smooth cap compatible with benign osteochondroma ( Fig. 1 ). However, sample size and location might determine a different diagnosis from the pathologist because they are difficult to differentiate. The histopathological analysis described by our pathologists is comparable to others that have been published. 1 After a review of the current literature on metachondromatosis, Fisher et al. found that 12 biopsies were studied; 42% (5/12) of the biopsies were diagnosed as osteochondromas, 33% (4/12) as enchondromas, and 17% (3/12) had multiple biopsies, some diagnosed as osteochondromas while some as enchondromas, as in our case. 1

### Treatment

Conservative treatment is the treatment of choice, because of the regressive potential and the near absence of malignization. 2 5 Metachondromatosis is an autosomal dominant disorder, so genetic advice must be given to patients. We recommend periodical monitoring of the lesions.

Surgical treatment is reserved for painful lesions: neurovascular compression (for example, equinus secondary to nervus fibularis communis compression in the peroneal head) and avascular necrosis of the femoral head. 1 4 5

Differential diagnosis between enchondromas, osteochondromas and metachondromatosis is vital due to differences in malignization and natural history. When a patient has multiple enchondromas and osteochondromas with regression of some lesions and osteochondroma-like lesions with atypical radiographical characteristics pointing toward the epiphysis, metachondromatosis, a rare disease, must be considered. Risk of malignization is insignificant and genetic advice must be given due it is an autosomal dominant disease.

## References

[1] Fisher T J Williams N Morris L Cundy P J Metachondromatosis: more than just multiple osteochondromas J Child Orthop 2013 7 06 455 464 24432109 10.1007/s11832-013-0526-3 PMC3886349

[2] McFarlane J Knight T Sinha A Cole T Kiely N Freeman R Exostoses, enchondromatosis and metachondromatosis; diagnosis and management Acta Orthop Belg 2016 82 01 102 105 26984661

[3] Wittram C Carty H Metachondromatosis Pediatr Radiol 1995 25 01 S138 S139 8577506

[4] Wenger D R Birch J Rathjen K Tobin R Billman G Metachondromatosis and avascular necrosis of the femoral head: a radiographic and histologic correlation J Pediatr Orthop 1991 11 03 294 300 2056076

[5] Mavrogenis A F Skarpidi E Papakonstantinou O Papagelopoulos P J Chondrosarcoma in metachondromatosis: a case report J Bone Joint Surg Am 2010 92 06 1507 1513 20516327 10.2106/JBJS.I.00693

[6] Pansuriya T C Kroon H M Bovée J V Enchondromatosis: insights on the different subtypes Int J Clin Exp Pathol 2010 3 06 557 569 20661403 PMC2907117

[7] Bowen M E Boyden E D Holm I A Loss-of-function mutations in PTPN11 cause metachondromatosis, but not Ollier disease or Maffucci syndrome PLoS Genet 2011 7 04 e1002050 21533187 10.1371/journal.pgen.1002050 PMC3077396

[8] Chun K A Stephanie S Choi J Y Nam J H Suh J S Enchondroma of the Foot J Foot Ankle Surg 2015 54 05 836 839 26024560 10.1053/j.jfas.2015.01.002

