# Supplementary material for: Metachondromatosis: A Confusing Disease
Source: Rev Bras Ortop (Sao Paulo). 2021 Nov 4;59(Suppl 1):e26–30. doi: 10.1055/s-0041-1736614 (PMC11254425; doi:10.1055/s-0041-1736614)
Supplement: Related texts

# Research article: Metacondromatose: Uma doença confusa

**Keywords:** criança, encondromas, exostose múltipla hereditária, neoplasias ósseas/patologia, osteocondromas

## Abstract

Metacondromatose é uma doença genética autossômica rara com penetração incompleta que envolve função anormal do gene *PTPN11* . A diferenciação entre tumores condrogênicos é um desafio para os ortopedistas. Relatamos um caso de uma menina de 5 anos com metacondromatose, doença que compartilha atributos com osteocondromas e encondromas. Encontramos múltiplas lesões semelhantes a osteocondromas com a característica atípica de guiar seu crescimento em direção à articulação vizinha (epífise) em vez de se afastar dela. Além disso, as lesões semelhantes a encondromas colunares eram claramente visíveis no raio distal direito, no colo uterino femoral proximal e nas cristas ilíacas. A paciente relatou que algum outro tumor tinha desaparecido ou reduzido com o tempo. Este caso foi debatido entre um grupo multidisciplinar de displasia esquelética. Os achados clínicos e radiográficos acima mencionados reforçam o diagnóstico hipotético da metacondromatose. O diagnóstico definitivo da metacondromatose é uma combinação de achados clínicos, radiográficos e histopatológicos. O diagnóstico diferencial entre encondromas, osteocondromas e metacondromatose é vital devido a diferenças na malignização e na história natural. Quando um paciente tem encondromas múltiplos e osteocondromas com regressão de algumas lesões e característica radiográfica atípica das lesões semelhantes ao osteocondroma apontando para a epífise, a metacondromatose, uma doença rara, deve ser considerada. Tratamento cirúrgico é reservado para lesões dolorosas. O risco de malignização é insignificante e conselhos genéticos devem ser dados por se tratar de uma doença autossômica dominante.

## Introdução

O osteocondroma é o tipo de tumor cartilaginoso benigno mais comum. Osteocondromas são tipicamente tumores metafiseais dos ossos longos (úmero proximal, tíbia e fêmur distal). Eles geralmente crescem como lesões pedunculadas ou séssil compostas de cortical e com tecido ósseo medular coberto por uma tampa cartilaginosa. 1

O encondroma é o segundo tipo de tumor cartilaginoso benigno mais comum após o osteocondroma. Eles são comumente encontrados dentro da cavidade medular dos ossos do esqueleto apendicular (mais frequentes nas mãos do que nos pés, em particular nas falanges) e são caracterizados pela formação de cartilagem hialina madura na cavidade medular. 2

A metacondromatose é uma doença genética autossômica rara com penetração incompleta que envolve função anormal do gene *PTPN11* . 1

A diferenciação entre tumores condrogênicos é um desafio para os ortopedistas. Relatamos o caso de uma paciente com metacondromatose, doença que compartilha atributos com osteocondromas e encondromas.

## Relato de Caso

Uma menina de 5 anos foi encaminhada para a Unidade de Ortopedia Pediátrica para avaliação de múltiplos osteocondromas. O exame físico revelou múltiplos tumores dolorosos compatíveis com osteocondromas na radiografia. Foi evidenciado prejuízo para flexão *proximal interphalangeal* (PIP) e *distal interphalangeal* (DIP) do quarto dedo da mão direita, que se correlacionava com um osteocondroma de falange média. O histórico anterior de doenças hereditárias esqueléticas não pôde ser confirmado. A excisão cirúrgica foi realizada sem complicações.

No entanto, o exame anatomopatológico mostrou múltiplas peças ósseas e cartilaginosas compatíveis com a capa externa de um encondroma benigno. Oito meses depois, ela também foi operada de um osteocondroma crescente e doloroso no terceiro metacarpo esquerdo e no quarto metacarpo direito. Paradoxalmente, neste caso, o exame anatomopatológico mostrou uma lesão óssea de 2,3 × 2 cm coberta com uma capa lisa branco-perolada compatível com osteocondroma benigno ( Fig. 1 ).

**Fig. 1:** Imagens de diferentes seções histológicas coloridas com hematoxilina-eosina. A) Amostra da lesão exostótica constituída por uma capa cartilaginosa com uma matriz osteoide central trabecular, 2,5X. B) A matriz condral apresenta características maduras com ossificação endocondral, 4X.C) Zona de transição entre o componente cartilaginoso periférico e osso trabecular semelhante a uma placa de crescimento ligeiramente desorganizada, 4X. D) Os condrócitos são dispostos em grupos isogênicos, maiores na porção central, sem atipia ou mitoses atípicas, 10X.

A radiografia anterior foi examinada para descobrir uma razão para este paradoxo inesperado. O que encontramos foram múltiplas lesões semelhantes a osteocondroma com a característica atípica de guiar seu crescimento em direção à articulação vizinha (epífise) em vez de se afastar dela. Além disso, as lesões semelhantes a encondromas colunares eram claramente visíveis no raio distal direito, no colo uterino femoral proximal e nas cristas ilíacas ( Figs. 2 e 3 ). A paciente relatou que algum outro tumor tinha desaparecido ou reduzido com o tempo. Este caso foi debatido entre um grupo multidisciplinar de displasia esquelética. Os achados clínicos e radiográficos acima mencionados reforçam o diagnóstico hipotético da metacondromatose.

**Fig. 2:** Visão anteroposterior de ambas as mãos mostrando múltiplas lesões semelhantes a osteocondroma com a característica atípica de guiar seu crescimento em direção à articulação vizinha (epífise) em vez de se afastar dela.

**Fig. 3:** Visão anteroposterior da pelve. Na metacondromatose, os encondromas se distribuem principalmente em torno da crista ilíaca e regiões metafisárias dos ossos longos.

Após 8 anos de acompanhamento, ela tem 13 anos, e novas lesões no tornozelo direito, quadril e falange média do quarto dedo da mão esquerda cresceram enquanto outras regrediram. No entanto, ela é assintomática e vive uma vida normal.

## Discussão

A metacondromatose combina múltiplas exostoses metafisárias justas, encondromas metafisários, calcificações periarticulares e frequentes alterações unilaterais ou bilaterais tipo Legg-Calvé-Perthes na cabeça femoral semelhantes a osteonecrose. 3 4 5

Classificação: a metacondromatose é um subtipo de encondromatose sem comprometimento espinhal, transmissão autossômica dominante e lesões semelhantes a osteocondromas. 6

Etiologia: metacondromatoses estão relacionadas com anormalidades genéticas. Fisher et al. encontraram 31 casos publicados. 1 A mutação do gene *PTPN11* (proteína tirosina fosfatase não-receptor tipo 11) e a falta de produção da fosfatase tirosina SHP2 estão relacionadas com a patogênese da metacondromatose, assim como de outras doenças do desenvolvimento (síndrome de Noonan, síndrome de Noonan com múltiplas lentiginas) e doenças malignas (leucemia mielomonocítica juvenil). 7 A mutação do gene *PTPN11* é herdada em um padrão autossômico dominante com penetração incompleta e os pais devem ser avisados disso. Ao contrário da encondromatose, a mutação EXT-1 e EXT-2 (proteína exostosina) não é observada na metacondromatose. 1 7

O diagnóstico definitivo da metacondromatose é uma combinação de achados clínicos, radiográficos e histopatológicos ( Tabela 1 ). 8

**Tabela 1:** Diagnóstico diferencial entre encondromas, osteocondromas e metacondromatose

	Encondromas	Osteocondromas	Metacondromatose
Frequência	10% de todos os tumores ósseos benignos. 8 A prevalência da doença de Ollier 1/100.000 1	20–50% de todos os tumores ósseos benignos e 10–15% de todos os tumores ósseos 7 Estima-se que a prevalência de osteocondromas múltiplos seja de 2/100.000 1	< 1/100.000.000, < 30 casos descritos 2
Localização	Frequentemente encontrados nas mãos mais do que nos ossos do pé e tornozelo; em particular nas falanges 1	Úmero proximal, tíbia e fêmur distal 1	Lesões semelhantes a encondroma: Regiões metafisárias dos ossos longos e crista ilíaca Lesões semelhantes a osteocondroma: são distribuídas principalmente nas mãos e nos pés 6
Genética	Não segue um padrão de transmissão mendeliano claro	HMO é um traço autossômico dominante herdado 7 Mutação EXT-1 e EXT-2 (proteína exostosina; transmembrana glicosiltransferase de retículo endoplasmático necessário para a síntese de sulfato heparan e crescimento fiseal)	Mutação genética *PTPN11* dominante autossômica, falta de fosfatase tirosina SHP2
Radiologia	Formação de cartilagem hialina na medula de um osso 2 Lesões líticas bem definidas, expansivas e com diferentes graus de calcificações pontilhadas ou pontuadas. Nas regiões diafisárias ou metafisárias-diafisárias do osso 1	Cartilagem pedunculada ou caroços sésseis fora da região metafisária dos ossos longos 2	Lesões semelhantes a osteocondroma de ponta epifisária combinadas com lesões calcificadas semelhantes a encondroma ( Figs. 1 e 2 ). Podem regredir espontaneamente 3
Exame anatomopatológico	Na inspeção visual bruta, um encondroma aparecerá como uma cartilagem de hialina azulada, semitranslúcida, com um arranjo claramente lobular. Estes lóbulos variam de alguns milímetros a alguns centímetros de diâmetro. Citologicamente, um encondroma aparecerá como pequenos condrócitos que estão nos espaços lacunares, com um núcleo pequeno, redondo, regular e sem atipia significativa. Nenhuma mitose será vista. Células binucleadas ocasionais serão vistas. Alguns encondromas podem conter focos de ossificação dentro desta cartilagem 1	Lesão óssea coberta com uma capa lisa branco-perolada	42% como osteocondromas, 33% como encondromas, 17% combinados 1
História natural	Novas lesões não aparecem após a maturação esquelética	Novas lesões não aparecem após a maturação esquelética	Novas lesões não aparecem após a maturação esquelética
Malignização	5% em encondromas solitários, > 20% encondromatose múltipla 1	Entre 0,4% e 2% em pacientes com osteocondroma solitário e entre 1 e 4% em pacientes com HMO 7	Nenhuma malignização

Achados clínicos: a combinação de encondromas múltiplos e osteocondromas levanta a suspeita de metacondromatose. 3 4 A metacondromatose tem lesões semelhantes a osteocondromas caracteristicamente epifisárias que podem regredir espontaneamente, em contraste com os osteocondromas convencionais. 3 4

Achados radiográficos: na metacondromatose, os encondromas se distribuem principalmente ao redor da cresta ilíaca e regiões metafisárias dos ossos longos ( Fig. 3 ). Em contraste, as lesões semelhantes a osteocondromas são distribuídas principalmente nas mãos e nos pés ( Fig. 2 ). 6 No nosso caso, vimos que essas lesões podem ser distribuídas tanto no esqueleto axial (pélvis, coluna, escápula, quadril) quanto no esqueleto apendicular (mãos e pés). As mãos foram as mais afetadas no nosso caso, em concordância com Fisher et al. 1 A metacondromatose não está relacionada com o encurtamento e a deformidade dos ossos longos, uma característica comum da exostose múltipla hereditária. 4 Assim como na osteocondromatose e na encondromatose, novas lesões não aparecem após a maturação esquelética. 1

Achados histopatológicos: o exame histopatológico relatou, primeiro, peças ósseas e cartilaginosas múltiplas compatíveis com a capa externa de um encondroma benigno e, depois, uma lesão óssea coberta com uma capa lisa branco-perolada compatível com osteocondroma benigno ( Fig. 1 ). No entanto, o tamanho e a localização da amostra podem determinar um diagnóstico diferente do patologista, pois são difíceis de diferenciar. A análise histopatológica descrita por nossos patologistas é comparável a outras publicadas. 1 Após revisão da literatura atual sobre metacondromatose, Fisher et al. descobriram que foram estudadas 12 biópsias; 42% (5/12) das biópsias foram diagnosticadas como osteocondromas, 33% (4/12) como encondromas e 17% (3/12) apresentaram biópsias múltiplas, algumas diagnosticadas como osteocondromas, enquanto algumas como encondromas, assim como o nosso caso. 1

## Tratamento

O tratamento conservador é o tratamento de escolha devido ao potencial regressivo e à quase ausência de malignização. 2 5 A metacondromatose é uma doença autossômica dominante, por isso devem ser dados conselhos genéticos aos pacientes. Recomendamos o monitoramento periódico das lesões.

O tratamento cirúrgico é reservado para lesões dolorosas: compressão neurovascular (ou sejam equinus secundário à compressão do nervus fibularis communis na cabeça peroneal) e necrose avascular da cabeça femoral. 1 4 5

O diagnóstico diferencial entre encondromas, osteocondromas e metacondromatose é vital devido a diferenças na malignização e na história natural. Quando um paciente tem encondromas múltiplos e osteocondromas com regressão de algumas lesões e característica radiográfica atípica das lesões semelhantes ao osteocondroma apontando para a epífise, a metacondromatose, uma doença rara, deve ser considerada. O risco de malignização é insignificante e conselhos genéticos devem ser dados devido à doença autossômica dominante.
